# Supplementary material for: Genomic and functional gene studies suggest a key role of beta-carotene oxygenase 1 like (bco1l) gene in salmon flesh color
Source: Sci Rep. 2019 Dec 27;9:20061. doi: 10.1038/s41598-019-56438-3 (PMC6934663; doi:10.1038/s41598-019-56438-3)
Supplement: Supplementary file 1 — Supplementary information [file 41598_2019_56438_MOESM1_ESM.pdf]

Supporting Online Material

Genomic and functional gene studies suggest a key role of *beta-carotene oxygenase 1 like (bco1l)* gene in salmon flesh color.

Hanna Helgeland[1,6+], Marte Sodeland[1,2,3+], Nina Zoric[1+], Jacob Seilø Torgersen[4], Fabian Grammes[1], Johannes von Lintig[5], Thomas Moen[4], Sissel Kjøglum[4], Sigbjørn Lien[1], Dag Inge Våge[1*].

[+] authors have contributed equally.

* corresponding author

[1] Centre for Integrative Genetics (CIGENE), Department of Animal and Aquacultural Sciences, Faculty of Biosciences, Norwegian University of Life Sciences, NO-1432 Ås, Norway.

[2]Institute of Marine research, N-4817 His, Norway

[3]University of Agder, Department of Natural Sciences, Faculty of Engineering and Science, N-4604 Kristiansand, Norway

[4]AquaGen, NO-7462 Trondheim, Norway.

[5]Case Western Reserve University, Department of Pharmacology, 2109 Adelbert Rd. Wood Bldg. 341, Cleveland, OH 44106, USA.

[6]Oslo University Hospital, Department of Radiation Biology, Institute for Cancer Research, N-0310 Oslo, Norway

FIGURE LEGENDS

Figure S1

Protein sequence alignment of BCO1 from human, mice, rat, cattle, sheep and zebrafish together with Salmo salar Bco1 and Bco1l. The four histidines coordinating the Fe^{2+} are shown in red letters. The additional residues making up the active site is highlighted with a black background, while the residues highlighted with a brown background are forming the patch located at the entrance to the active tunnel. The sequences are: NP_059125.2 (*Homo sapiens*), NP_001019730.1 (*Bos taurus*), NP_001295500.1 (*Ovis aries*), NP_067461.2 (*Mus musculus*), NP_446100.2 (*Rattus norvegicus*), NP_001315424.1 (*Danio rerio*), NP_001266000.1 (*Salmo salar - bco1*), NP_001266003.1 (*Salmo salar - bco1l*)

Figure S2

(A) Overlay cartoon view of predicted and spatially aligned Atlantic salmon Bco1 (purple) and Bco1l (cyan) structures, modeled with SWISS-MODEL program based on related RPE65 template 3fsn.1A (2.14 Å). (B) The iron cofactor, shown as an orange sphere, is directly coordinated by four histidine residues. (C) Iron cofactor with all the surrounding amino acid residues. For viewing PyMol was used.

Figure S3

Regions of misalignment between predicted 3D Bco1 and Bco1l models.

	1	10	20	30	40
*Homo sapiens*_BCO1	~MDIIFGRNR	KEQLEPVRAK	VTGKIPAWLQ	GTLLRNGPGM	HTVGESRYNH
*Bos taurus*_BCO1	~MEIIFGRNK	KEQLEPVRAR	VTGKIPAWLQ	GILLRNGPGM	HTVGETRYNH
*Ovis aries*_BCO1	~MEIIFGRNK	KEQLEPVRAR	VTGKIPAWLQ	GTLLRNGPGM	HTVGETRYNH
*Mus musculus*_BCO1	~MEIIFGQNK	KEQLEPVQAK	VTGSIPAWLQ	GTLLRNGPGM	HTVGESKYNH
*Rattus norvegicus*_BCO1	~MEIIFGRNK	KEQLEPLRAT	VTGSIPAWLQ	GTLLRNGPGM	HTVGDSKYNH
*Danio rerio*_BCO1	~MQYDYGKNK	EEHPEPIKTE	VKGSIPEWVQ	GTLIRNGPGM	FSVGETTYNH
Salmo salar_BCO1	~MAYDYAKNR	EERPDPVKAD	LKGNLPSWLQ	GTLLRNGPGI	FSVGDTTYNH
Salmo salar_BCO1l	MSQSLIGKNG	TESPEPVKAE	VTGCVPEWLQ	GTLLRNGPGL	FNVGATEYNH

	50	60	70	80	90
*Homo sapiens*_BCO1	WFDGLALLHS	FTIRDGEVYY	RSKYLRSDTY	NTNIEANRIV	VSEFGTMAYP
*Bos taurus*_BCO1	WFDGLALLHS	FTIRDGEVYY	RSKYLRSDTY	TANIEANRIV	VSEFGTMAYP
*Ovis aries*_BCO1	WFDGLALLHS	FTIRDGEVYY	RSKYLRSDTY	TANIEANRIV	VSEFGTMAYP
*Mus musculus*_BCO1	WFDGLALLHS	FSIRDGEVFY	RSKYLQSDTY	IANIEANRIV	VSEFGTMAYP
*Rattus norvegicus*_BCO1	WFDGLALLHS	FSIRDGEVFY	RSKYLQSDTY	NANIEANRIV	VSEFGTMAYP
*Danio rerio*_BCO1	WFDGMALLHS	FAINKGEVTY	RSRYLRGDTY	NSNMQANRIV	VSEMGTMAYP
Salmo salar_BCO1	WFDGMALMHS	FTFKDGEVIY	RSRYLRGDTY	KDNMAAKRIV	VSEMGTMAYP
Salmo salar_BCO1l	WFDGMALIHS	FTFKDGEVYY	RSKFLRSDTF	KKNTQANKIV	VSEFGTMIYP

	100	110	120	130	140
*Homo sapiens*_BCO1	DPCKNIFSKA	FSYLSHTIPD	FTDNCLINIM	KCGEDFYATS	ETNYIRKINP
*Bos taurus*_BCO1	DPCKNIFSKA	FSYLSHTIPD	FTDNCLINIR	RCGEDFYATT	ETSYIRRINP
*Ovis aries*_BCO1	DPCKNIFSKA	FSYLSHTIPD	FTDNCLINIM	RCGEDFYATT	ETNYIRKINP
*Mus musculus*_BCO1	DPCKNIFSKA	FSYLSHTIPD	FTDNCLINIM	KCGEDFYATT	ETNYIRKIDP
*Rattus norvegicus*_BCO1	DPCKNIFSKA	FSYLSHTIPD	FTDNCLINIM	KCGEDFYATT	ETNYIRKIDP
*Danio rerio*_BCO1	DPCKNIFSKV	ITFLSHTIPD	FTDNCGNNII	KYGNDFHATS	ETNYIRKIDP
Salmo salar_BCO1	DPGKNVISRV	ITFLNHTVPD	FTDNCGNNFI	RYGKDYYATS	ETNYIRKVDP
Salmo salar_BCO1l	DPCKNIFSKA	FSYLLAAIPD	FTDNNLINII	RYGEDYYASS	EVNYMNQIDP

	150	160	170	180	190
*Homo sapiens*_BCO1	QTLETLEKVD	YRKYVAVNLA	TSHPHYDEAG	NVLNMGTSIV	EKGKTKYVIF
*Bos taurus*_BCO1	QTLETLEKVD	FRKYVAVNLA	TSHPHYDAAG	NVLNVGTSIV	DKGKTKYVIF
*Ovis aries*_BCO1	QTLETLEKVD	YRKYVAVNLA	TSHPHYDAAG	NVLNVGTSIV	DKGKTKYVIF

*Mus musculus*_BCO1	QTLETLEKVD	YRKYVAVNLA	TSHPHYDEAG	NVLNMGTSVV	DKGRTKYVIF
*Rattus norvegicus*_BCO1	QTLETLEKVD	YRKYVAVNLA	TSHPHYDEAG	NVLNMGTSIA	DKGRTKYVMF
*Danio rerio*_BCO1	VTLETQEKID	YLKYLPVSIV	ASHTHYDKEG	NSYSMGTCIA	EKGKTKYMLF
Salmo salar_BCO1	VTLETQDKVD	YMKYLAVNLV	TSHPHYDKDG	TAYNIGTSIA	EKGKTKYTLF
Salmo salar_BCO1l	MTLDVIGKMN	YRNHIALNMA	TAHPHYDDEG	NTYNMGTALM	RFGMPNYVIF
	200	210	220	230	240
*Homo sapiens*_BCO1	KIPATVPEGK	KQGKSPWKHT	EVFCSIPSRS	LLSPSYYHSF	GVTENYVIFL
*Bos taurus*_BCO1	KIPAPVPGGR	KEGRSPLKDT	EVFCSIAAHS	LLSPSYYHSF	GVSENYIIFL
*Ovis aries*_BCO1	KIPATVPGGR	KEGRSPLKDA	EVFCSIAARS	LLSPSYYHSF	GVTENYVVFL
*Mus musculus*_BCO1	KIPATVPDSK	KKGKSPVKHA	EVFCSISSRS	LLSPSYYHSF	GVTENYVVFL
*Rattus norvegicus*_BCO1	KIPATAPGSK	KKGKNPLKHS	EVFCSIPSRS	LLSPSYYHSF	GVTENYVVFL
*Danio rerio*_BCO1	KVPG...ESR	PDGSPPLKSA	EAVCTLPCRS	LLTPSYYHSF	GMTDNYFIFI
Salmo salar_BCO1	KVPDTTAGDK	ANASPALKNL	EVICTVPCRS	LLSPSYYHSF	GMTDNYLIFI
Salmo salar_BCO1l	KVPVNA.SDK	EHKKPALRKV	KQVCNIPIRS	TLFPSYFHSF	GMTENYIIFV
	250	260	270	280	290
*Homo sapiens*_BCO1	EQPFRLDILK	MATAYIRRMS	WASCLAFHRE	EKTYIHIIDQ	RTRQPVQTKF
*Bos taurus*_BCO1	EQPFKLDILK	MATAYIRGVS	WASCLAFHGE	DKTHIHIIDR	RTRKPVPTKY
*Ovis aries*_BCO1	EQPFKLDILK	MATAYIRGVS	WASCLAFHGE	DKTHIHIIDR	RTRKPVLAKY
*Mus musculus*_BCO1	EQPFKLDILK	MATAYMRGVS	WASCMSFDRE	DKTYIHIIDQ	RTRKPVPTKF
*Rattus norvegicus*_BCO1	EQPFKLDILK	MATAYMRGVS	WASCMTFCKE	DKTYIHIIDQ	KTRKPVPTKF
*Danio rerio*_BCO1	EQPLKLDILK	MATAYLRRVS	WASCMKFHPE	DSTLIHLIDR	NTKKEVATKF
Salmo salar_BCO1	EQPFKLDILK	MATAYMRGVN	WASCLKFCPE	ENTLIHLIDR	KTGKEVGIKY
Salmo salar_BCO1l	EQPFKLDILR	LATAIFRRVT	WASCLKYDKE	DITLIHLIDK	KTGKAVSTKF
	300	310	320	330	340
*Homo sapiens*_BCO1	YTDAMVVFHH	VNAYEEDGCI	VFDVIAYEDN	SLYQLFYLAN	LNQDFKE...
*Bos taurus*_BCO1	HTDPMVVFHH	VNAYEEDGCL	LFDVITYEDG	SLYQLFYLAN	LNEDFKE...
*Ovis aries*_BCO1	HTDPMVVFHH	VNAYEEDGCL	LFDVIAYEDG	SLYQLFYLAN	LNEDFKE...
*Mus musculus*_BCO1	YTDPMVVFHH	VNAYEEDGCV	LFDVIAYEDS	SLYQLFYLAN	LNKDFEE...
*Rattus norvegicus*_BCO1	YTDPMVVFHH	VNAYEEDGCV	LFDVIAYEDN	SLYQLFYLAN	LNKDFEE...
*Danio rerio*_BCO1	YTDAMTVYHQ	VNAFEDDGHV	VFDVIAYDDN	NLYEFFYLNK	LKETMGA...
Salmo salar_BCO1	YTEAMIVYHH	VNAFEEDGHV	IFDVIAYEDP	SLYNMFYLNV	LKEQSKA...
Salmo salar_BCO1l	YTDALVVFHH	INAYEDDGHV	VFDMITYKDG	NLYEMFYFAN	LRKETQEFIE

		356	366	376	386
*Homo sapiens*_BCO1	.NSRLTSVPT	LRRFAVPLHV	DKNAEVGTNL	IKVASTTATA	LKEEDGQVYC
*Bos taurus*_BCO1	.NSRLTSMPT	LKRFVLPLHV	DKNAEVGSNL	IKLSSTTARA	LKEKDDQVYC
*Ovis aries*_BCO1	.NSRLTSMPT	LKRFVLPLHV	DKNAEVGSNL	INLSSTTARA	LKEKDGQVYC
*Mus musculus*_BCO1	.KSRLTSVPT	LRRFAVPLHV	DKDAEVGSNL	VKVSSTTATA	LKEKDGHVYC
*Rattus norvegicus*_BCO1	.KSRLTSVPT	LRRFAVPLHV	DKDAEVGSNL	VKVSSTTATA	LKEKDDHVYC
*Danio rerio*_BCO1	..TNLYCKPK	FTRFVFPL.S	DQG.ETGENL	VKLKYTTASA	VKEKDGKIMC
Salmo salar_BCO1	...SAMSVPK	CKRFALPVQN	DKGIDVGDDM	VKLQYTTASA	VKEKEGKLLC
Salmo salar_BCO1l	SNKVNISPPI	CQRFVLPLTV	DKDTSNGTNL	VRLKDTTAKA	VMQSDGSLYC
	396	406	416	426	436
*Homo sapiens*_BCO1	QPEFLYEGLE	LPRVNYAHNG	KQYRYVFATG	VQWSPIPTKI	IKYDILTKSS
*Bos taurus*_BCO1	QPELLCEGLE	LPHINYAHNG	QPYRYIFAAG	VQWSPRPLIY	AAIR.LAKSS
*Ovis aries*_BCO1	QPELLYEGLE	LPRINYAHNG	KPYRYVFAAG	VQWSPIPTQI	IKYDILTKSS
*Mus musculus*_BCO1	QPEVLYEGLE	LPRINYAYNG	KPYRYIFAAE	VQWSPVPTKI	LKYDILTKSS
*Rattus norvegicus*_BCO1	QPEVLYEGLE	LPRINYAHNG	KPYRYIFAAE	VQWSPVPTKI	LKYDVLTKSS
*Danio rerio*_BCO1	QGEVLCEGVE	LPRINYNFNG	KKYRYSYMCC	VDESPVATRI	VKFDADTKQQ
Salmo salar_BCO1	QPEVLCDGVE	LPRINYDFNG	KKYRFVYMTC	VAMSAVATKI	MKLDTETKER
Salmo salar_BCO1l	LPETIFQGLE	LPGMNYKFNG	KKYRYFYGSR	VEWTPHPNKI	GKVDIVTRKY
	446	456	466	476	486
*Homo sapiens*_BCO1	LKWREDDCWP	AEPLFVPAPG	AKDEDDGVIL	SAIVSTDPQK	LPFLLILDAK
*Bos taurus*_BCO1	LTWKEEHCWP	AEPLFVPTPG	AKDEDDGIIL	SAIVSTDPQK	SPFLLVLDAR
*Ovis aries*_BCO1	LKWGEEHCWP	AEPLFVPTPG	AKDEDDGIIL	SAIVSTDPQK	SPFLLVLDAR
*Mus musculus*_BCO1	LKWSEESCWP	AEPLFVPTPG	AKDEDDGVIL	SAIVSTDPQK	LPFLLILDAK
*Rattus norvegicus*_BCO1	LKWSEESCWP	AEPLFVPTPG	AKDEDDGVIL	SAIISTDPQK	LPFLLILDAK
*Danio rerio*_BCO1	IEWKGDDGFA	SEPVFIPRPG	AVDEDDGVVL	TVIINNKPLQ	GGFLLVLDAK
Salmo salar_BCO1	TEWREENCWP	SEPVFIPRPN	GEGEDDGVVL	TTVINSNPGE	SGFILVLDAK
Salmo salar_BCO1l	IEWTEKDCYP	SEPVFVASPG	AVEEDDGVIL	TSVVSLNPKK	SPFMLVLNAK
	496	506	516	526	536
*Homo sapiens*_BCO1	SFTELARASV	DVDMHMDLHG	LFITDMDWDT	KKQAASEEQR	DRASDCHGAP
*Bos taurus*_BCO1	TFTELARASV	DVEMHLDFHG	LFIPDAGRDP	GKQAPSQEAP	ARAAAGRAAP
*Ovis aries*_BCO1	TFTELARASI	DVEMHLDIHG	LFIPDAGWDL	GKQAPSREAP	ARAAAGRAAP
*Mus musculus*_BCO1	SFTELARASV	DADMHLDLHG	LFIPDADWNA	VKQTPAETQE	VENSDHPTDP

```
Rattus norvegicus_BCO1 SFTELARASV DVDMHLDLHG LFIPDAGWNA VKQTPAKTQE DENSDHPTGL
Danio rerio_BCO1       SFKEIARACL DVEIHMDMHG YFIPGSS~~~ ~~~~~~~~~~ ~~~~~~~~~~
Salmo salar_BCO1       SFKEVARAHV NAELHMDMHG YFIPMEN~~~ ~~~~~~~~~~ ~~~~~~~~~~
Salmo salar_BCO1l      TFEEIARASI DASIHLDLHG HFIPTQSTN~ ~~~~~~~~~~ ~~~~~~~~~~

                       546
Homo sapiens_BCO1      LT~~~~~~~~ ~~~~~~~~~~ ~~~~~~~~~~ ~~~~~~~~~~ ~~~~~~~~~~
Bos taurus_BCO1        RTDSLEALVL GTSSAQLTAV PAPGEGRESG PSFHFAHILS AAALSQNSET
Ovis aries_BCO1        QT~~~~~~~~ ~~~~~~~~~~ ~~~~~~~~~~ ~~~~~~~~~~ ~~~~~~~~~~
Mus musculus_BCO1      TA........ .......... PELSHSENDF TAGHGGSSL~ ~~~~~~~~~~
Rattus norvegicus_BCO1 TA........ .......... PGLGHGENDF TAGHGGKSL~ ~~~~~~~~~~
Danio rerio_BCO1       ~~~~~~~~~~ ~~~~~~~~~~ ~~~~~~~~~~ ~~~~~~~~~~ ~~~~~~~~~~
Salmo salar_BCO1       ~~~~~~~~~~ ~~~~~~~~~~ ~~~~~~~~~~ ~~~~~~~~~~ ~~~~~~~~~~
Salmo salar_BCO1l      ~~~~~~~~~~ ~~~~~~~~~~ ~~~~~~~~~~ ~~~~~~~~~~ ~~~~~~~~~~

Homo sapiens_BCO1      ~~
Bos taurus_BCO1        ET
Ovis aries_BCO1        ~~
Mus musculus_BCO1      ~~
Rattus norvegicus_BCO1 ~~
Danio rerio_BCO1       ~~
Salmo salar_BCO1       ~~
Salmo salar_BCO1l      ~~
```

Figur S1 - Helgeland et al.

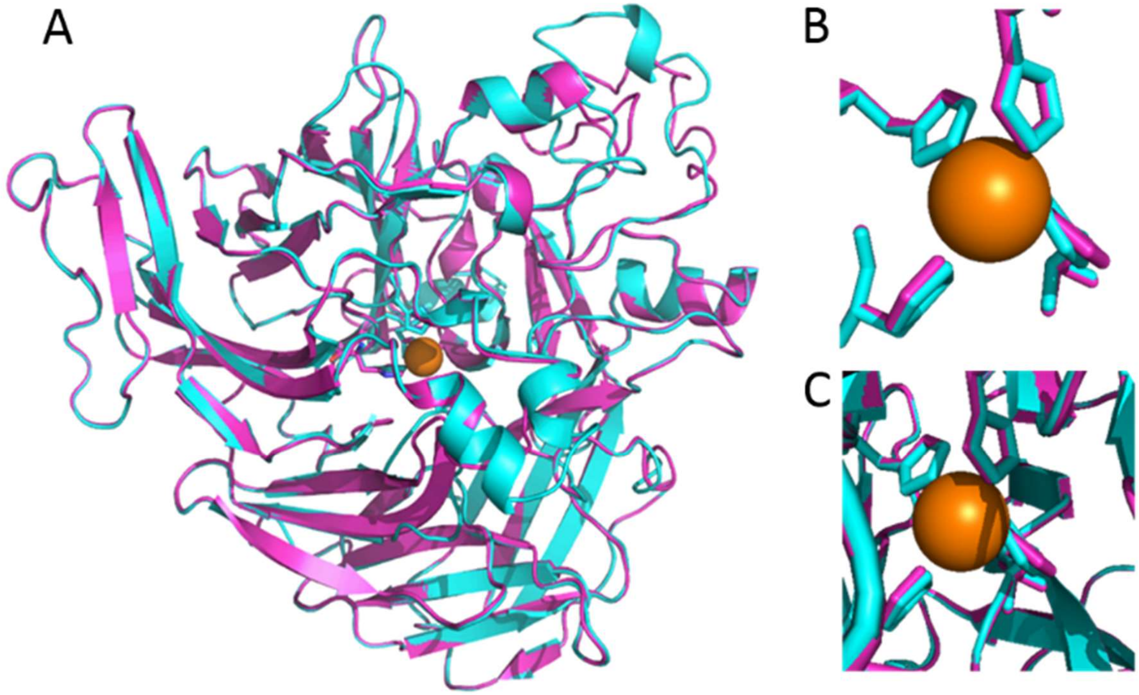

Figure S2 - Helgeland et al.

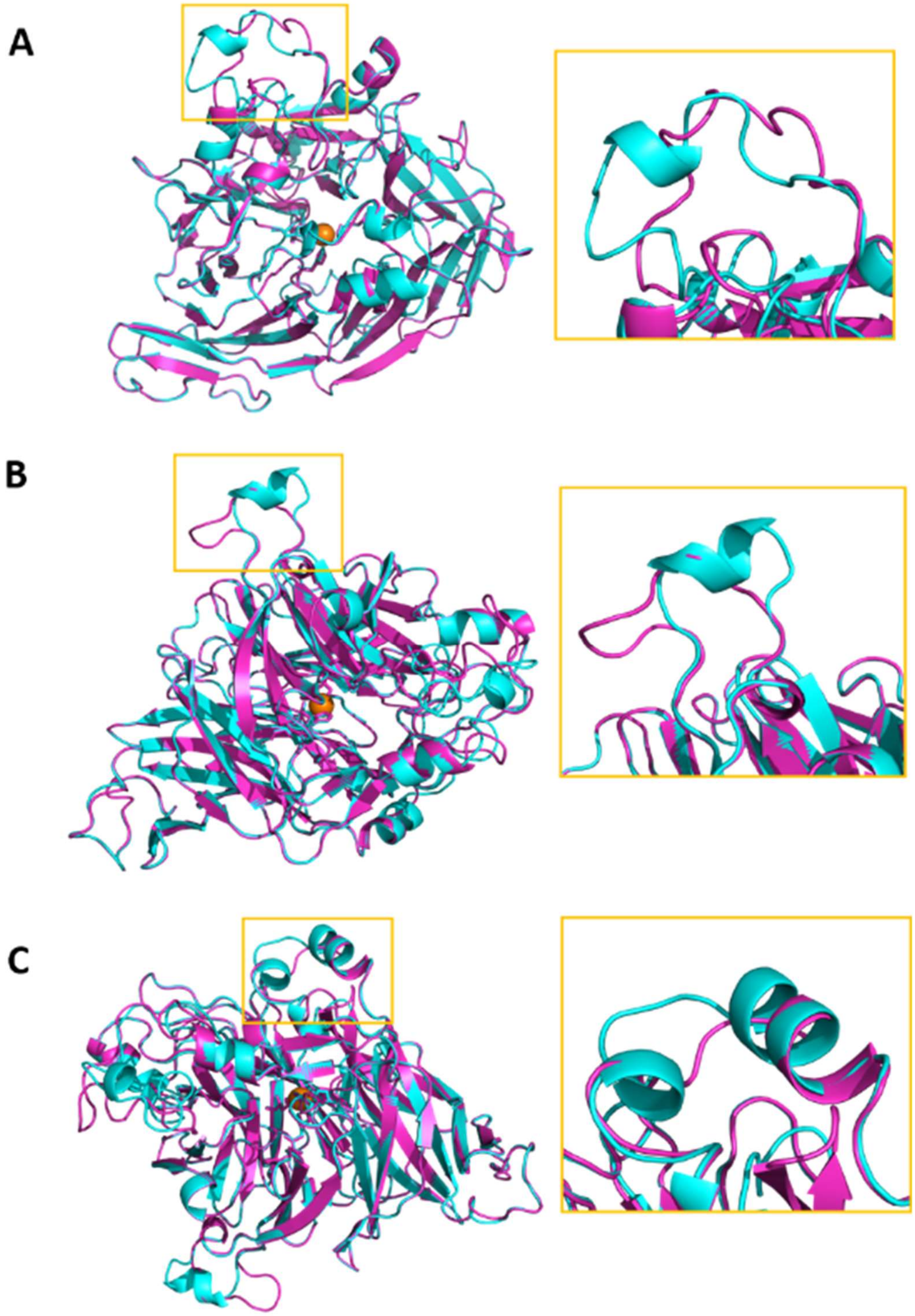

Figure S3 - Helgeland et al.

Table S1

SNP-identity, positions (assembly ICSASG_v2) and p-values for all the SNPs included in the fine-mapping at Ssa26. The SNPs without a rs-number has been submitted to European Variation Archive (EVA) under Project: PRJEB28258.

SNP_ID	position	P-value	LogP
ctg7180001912552_1401_SCT	18165496	1.330E-07	15.83
rs863643757	18209026	1.563E-08	17.97
rs863630607	18209503	4.376E-08	16.94
rs864131335	18212528	3.846E-03	5.56
rs863929219	18212572	1.229E-05	11.31
rs863994564	18214352	7.186E-01	0.33
rs863246859	18214880	4.850E-01	0.72
rs863302789	18216152	5.767E-02	2.85
rs864216165	18216446	5.725E-02	2.86
rs864189383	18216795	5.767E-02	2.85
rs864076812	18217233	9.143E-03	4.69
rs863526953	18217539	5.725E-02	2.86
rs863923796	18219658	5.767E-02	2.85
rs863854450	18220244	5.767E-02	2.85
rs863370817	18220852	6.305E-01	0.46
rs863758464	18220868	6.305E-01	0.46
rs863751717	18225298	5.192E-02	2.96
rs863472796	18226558	5.811E-01	0.54
rs863870255	18245647	1.128E-02	4.48
rs863670640	18249825	3.691E-01	1.00
rs863942373	18250502	7.280E-01	0.32
rs863848334	18250708	5.951E-01	0.52
rs863358817	18252043	5.246E-11	23.67
rs863749263	18254032	7.606E-05	9.48
rs863527645	18254565	6.628E-02	2.71
rs863742312	18254876	6.628E-02	2.71
rs863875634	18255060	3.691E-01	1.00
rs864212792	18255144	2.818E-04	8.17
rs863445911	18255574	2.097E-04	8.47
rs863279327	18255833	3.450E-04	7.97
rs863668595	18255912	1.100E-11	25.23
rs863405534	18256181	4.237E-04	7.77
rs863678353	18257349	9.904E-13	27.64
rs864011705	18257454	9.904E-13	27.64

rs863268852	18259051	6.802E-02	2.69
rs864146797	18278051	6.598E-01	0.42
rs864022302	18281310	1.424E-02	4.25
rs863861608	18285252	1.222E-06	13.61
rs863645922	18285350	3.452E-07	14.88
rs864159650	18286456	3.244E-01	1.13
rs864062338	18286948	1.084E-02	4.52
rs863545505	18287102	8.816E-01	0.13
rs863742875	18287250	8.816E-01	0.13
rs864214566	18287506	3.691E-01	1.00
rs863530134	18287701	3.691E-01	1.00
rs863610917	18289359	3.421E-06	12.59
rs863809717	18297483	1.215E-03	6.71
ctg7180001937482_1652_SAG	18300048	9.065E-01	0.10
rs863570483	18330028	6.889E-01	0.37
rs863460169	18332201	6.807E-04	7.29
rs864222221	18332544	1.388E-02	4.28
rs863239536	18339421	2.704E-01	1.31
rs863261398	18344217	2.225E-03	6.11
rs864045796	18344416	2.225E-03	6.11
rs863467318	18347696	2.225E-03	6.11
rs863848265	18352177	3.486E-01	1.05
rs863951906	18357758	1.298E-02	4.34
rs863883327	18366158	1.138E-02	4.48
rs863409857	18367282	9.249E-02	2.38
rs863756163	18369434	9.249E-02	2.38
rs863621526	18372945	1.173E-01	2.14
rs863257497	18373077	1.173E-01	2.14
rs863930762	18374240	1.173E-01	2.14
rs863955152	18374323	5.533E-02	2.89
rs863270876	18375356	1.703E-01	1.77
rs864226610	18375430	6.788E-02	2.69
rs863405630	18376136	1.704E-01	1.77
rs863490287	18376340	2.978E-02	3.51
rs864231524	18376749	2.978E-02	3.51
rs863551430	18380372	3.378E-04	7.99
rs863245462	18383378	9.139E-02	2.39
rs863242518	18383929	5.039E-01	0.69
rs863504373	18385547	6.855E-01	0.38
rs864004026	18396882	5.578E-01	0.58
ctg7180001910618_6988_SAG	18397478	7.997E-01	0.22
rs863904970	18400408	3.476E-01	1.06
rs863883651	18400771	2.341E-05	10.66

rs863655491	18401061	3.476E-01	1.06
rs863336140	18402164	8.303E-06	11.70
rs863827406	18407360	1.844E-01	1.69
ctg7180001934204_510_SAG	18411761	4.158E-03	5.48
rs864211565	18412415	6.664E-02	2.71
ctg7180001934204_1641_SAG	18412892	3.667E-05	10.21
rs863739786	18414556	3.844E-03	5.56
rs159401219	18414602	1.621E-06	13.33
rs159401218	18414949	4.128E-02	3.19
rs863879353	18415545	1.091E-02	4.52
rs864113451	18415688	9.424E-10	20.78
rs863601299	18415730	3.001E-01	1.20
rs863898337	18415786	5.117E-13	28.30
rs863905018	18422579	3.542E-01	1.04
rs864138807	18423344	5.101E-04	7.58
rs864120442	18424569	9.743E-04	6.93
rs863373926	18426927	1.318E-02	4.33
rs863348554	18427812	2.370E-02	3.74
ctg7180001866800_6544_SAG	18429158	7.757E-01	0.25
rs863402331	18432173	7.470E-08	16.41
rs864029623	18432240	3.006E-01	1.20
rs863661748	18432582	1.693E-07	15.59
rs863711429	18432785	6.071E-09	18.92
rs863291822	18432840	1.527E-03	6.48
rs863564784	18432868	3.015E-01	1.20
rs863267812	18433531	3.015E-01	1.20
rs863746472	18433823	1.331E-04	8.92
rs863419768	18433908	1.691E-04	8.69
rs863235361	18433914	1.691E-04	8.69
rs863315971	18434126	1.029E-07	16.09
rs863274339	18434589	1.691E-04	8.69
ctg7180001934204_23777_SAG	18435029	5.895E-02	2.83
rs864091273	18435996	4.530E-09	19.21
rs863689986	18436349	4.820E-02	3.03
rs863933324	18436862	7.296E-01	0.32
rs864043929	18440020	2.569E-01	1.36
rs863746292	18440590	2.569E-01	1.36
rs864000441	18441078	2.569E-01	1.36
rs863630746	18441401	3.414E-02	3.38
rs863740261	18443283	2.569E-01	1.36
rs863974888	18447307	1.341E-04	8.92
rs863470605	18447523	2.569E-01	1.36
rs864216526	18449231	5.939E-01	0.52

ctg7180001841303_5259_SAG	18457743	1.005E-02	4.60
rs863337839	18459452	2.824E-01	1.26
rs864145384	18460914	1.389E-06	13.49
rs863967569	18461912	6.130E-02	2.79
ctg7180001352914_8470_SGT	18467059	9.203E-07	13.90
rs863996077	18467277	1.128E-07	16.00
ctg7180001352914_9263_SAG	18467852	8.782E-05	9.34
rs863921815	18479105	3.013E-01	1.20
rs863707711	18479426	3.013E-01	1.20
rs863536299	18485339	3.029E-03	5.80
rs863473627	18487418	8.131E-11	23.23
rs863324020	18487479	5.521E-02	2.90
rs863521503	18488034	7.304E-02	2.62
rs864141582	18490224	4.953E-04	7.61
rs863934226	18492896	6.967E-06	11.87
rs863584931	18504183	2.969E-02	3.52
rs863768234	18504237	1.259E-04	8.98
rs863854470	18504285	4.283E-02	3.15
rs864081920	18506731	2.218E-04	8.41
rs863704000	18508611	2.218E-04	8.41
rs864075902	18514528	2.218E-04	8.41
rs863585735	18514538	2.218E-04	8.41
rs863454153	18518899	1.259E-04	8.98
rs863606098	18522330	2.909E-04	8.14
rs863247952	18522988	2.836E-02	3.56
rs863518137	18523082	9.840E-01	0.02
rs863670279	18524388	3.036E-02	3.49
rs864180255	18525089	6.465E-01	0.44
rs863525606	18525582	4.475E-04	7.71
rs863597426	18530653	6.698E-02	2.70
rs864066718	18531084	8.904E-01	0.12
rs863232239	18532566	8.904E-01	0.12
rs863633278	18533037	8.969E-03	4.71
rs863848436	18533819	8.232E-06	11.71
rs863786003	18534062	7.277E-01	0.32
ctg7180001805095_6306_SAC	18542907	1.463E-01	1.92
rs863695107	18547947	5.617E-02	2.88
rs863325783	18548016	6.456E-01	0.44
rs863967251	18549631	4.535E-02	3.09
rs863870456	18550971	5.803E-05	9.75
rs864052330	18551004	5.803E-05	9.75
rs863879095	18554315	7.325E-01	0.31
rs863866399	18555608	7.325E-01	0.31

rs863603382	18556374	6.253E-01	0.47
rs863521067	18569929	7.655E-02	2.57
rs864180301	18573106	1.937E-05	10.85
rs863428566	18579728	4.974E-07	14.51
rs863629571	18579732	6.780E-05	9.60
rs863512936	18581586	1.502E-03	6.50
rs864142918	18583319	2.591E-03	5.96
rs863432906	18583940	6.471E-01	0.44
rs863823788	18584663	6.471E-01	0.44
rs863565691	18586336	3.828E-05	10.17
rs863562206	18586597	4.954E-01	0.70
rs863815715	18599967	2.483E-06	12.91
rs863749953	18600666	6.667E-01	0.41
rs863483118	18600892	2.659E-08	17.44
rs863294192	18601824	2.659E-08	17.44
rs863372498	18607960	5.428E-02	2.91
rs864247432	18608983	2.659E-08	17.44
rs863470433	18609040	2.659E-08	17.44
rs863799208	18609181	2.659E-08	17.44
rs863354021	18619054	2.154E-02	3.84
rs864228317	18622171	4.925E-01	0.71
rs864176083	18622336	2.209E-01	1.51
rs864083818	18622816	6.213E-04	7.38
rs863257025	18622998	4.552E-06	12.30
rs863276828	18624465	1.828E-02	4.00
rs864022708	18624688	2.452E-02	3.71
ctg7180001779364_1565_SCT	18624729	1.231E-02	4.40
ctg7180001860735_7383_SAG	18624863	2.138E-02	3.85
rs863438371	18625071	2.152E-07	15.35
rs863734473	18625261	3.586E-05	10.24
rs864057710	18625584	2.467E-03	6.00
rs863659014	18627218	1.150E-06	13.68
rs863737351	18627328	3.586E-05	10.24
rs864074323	18627815	3.754E-01	0.98
rs864063606	18628106	1.744E-05	10.96
rs863607845	18629160	3.754E-01	0.98
rs863365385	18629283	3.680E-07	14.82
rs864109729	18634188	6.154E-06	12.00
ctg7180001334602_552_SCT	18635466	5.000E-01	0.69
rs863457882	18637926	6.154E-06	12.00
rs864036713	18638069	2.659E-08	17.44
rs863427677	18638690	2.659E-08	17.44
rs863498848	18638885	1.439E-01	1.94

rs863293399	18643466	6.714E-01	0.40
rs863664652	18643877	7.235E-09	18.74
rs863900687	18644168	3.080E-06	12.69
rs864243805	18644202	2.659E-08	17.44
rs864105311	18644640	2.659E-08	17.44
rs863740510	18648223	2.659E-08	17.44
rs863308265	18648722	4.132E-03	5.49
rs863795279	18648849	2.659E-08	17.44
rs863422146	18649630	2.659E-08	17.44
rs863956787	18649744	2.659E-08	17.44
rs863538184	18649792	1.127E-01	2.18
rs864031980	18650133	1.341E-07	15.82
rs863418676	18670222	1.270E-04	8.97
rs863252336	18670508	6.560E-01	0.42
rs863883261	18671558	1.879E-01	1.67
rs864119264	18672365	1.879E-01	1.67
rs863346645	18672938	1.154E-07	15.97
ctg7180001901066_4965_SAT	18673463	8.571E-06	11.67
rs864224668	18675116	2.307E-04	8.37
rs863946442	18675437	2.362E-05	10.65
rs863373340	18675525	2.860E-04	8.16
rs864230082	18675920	2.659E-08	17.44
rs864087201	18676038	2.659E-08	17.44
rs863787462	18676433	7.306E-03	4.92
rs863838924	18676485	6.563E-05	9.63
rs863639625	18677056	1.816E-04	8.61
rs863734750	18677555	1.909E-05	10.87
rs863264291	18678887	1.290E-07	15.86
rs863710116	18678948	1.356E-02	4.30
rs863850827	18679128	1.356E-02	4.30
rs863401956	18690950	2.495E-06	12.90
rs863860980	18694935	5.355E-02	2.93
rs864229410	18695400	6.317E-01	0.46
rs864097358	18710902	2.000E-08	17.73
rs863568638	18710943	1.869E-05	10.89
rs863772437	18711924	8.813E-01	0.13
rs863408412	18712894	8.813E-01	0.13
rs864191959	18713145	8.813E-01	0.13
rs863747597	18715000	8.813E-01	0.13
rs863697357	18715771	2.698E-03	5.92
rs863775212	18716559	2.659E-08	17.44
rs864187056	18716576	2.659E-08	17.44
rs863525292	18717095	5.745E-01	0.55

rs864064286	18717423	3.412E-06	12.59
rs863661189	18717608	3.996E-05	10.13
ctg7180001895681_5855_SAC	18718235	9.121E-01	0.09
rs863874948	18718773	5.959E-08	16.64
rs864156042	18720538	5.110E-01	0.67
rs864226477	18720593	5.110E-01	0.67
rs863860690	18725688	5.469E-02	2.91
rs863333509	18729684	9.274E-01	0.08
rs863850490	18733202	2.659E-08	17.44
rs864152410	18737057	2.659E-08	17.44
rs863241295	18738280	2.443E-03	6.01
rs864207151	18738817	2.443E-03	6.01
rs863664674	18738880	1.799E-08	17.83
rs864225571	18739096	1.412E-01	1.96
rs864124247	18739626	6.994E-01	0.36
rs863983453	18739795	3.169E-04	8.06
rs863243102	18740287	3.767E-01	0.98
rs863781128	18740394	4.560E-04	7.69
ctg7180001340274_15139_SGT	18740601	9.609E-01	0.04
rs863788280	18741600	9.601E-01	0.04
rs864222418	18742780	1.908E-07	15.47
rs863406367	18742930	8.948E-01	0.11
rs863652269	18742958	1.386E-07	15.79
rs863737359	18743190	1.725E-04	8.67
rs864185737	18743261	1.725E-04	8.67
rs863704627	18743932	1.412E-04	8.87
rs863534823	18744097	1.412E-04	8.87
rs863302884	18744580	8.516E-01	0.16
rs863750215	18745576	1.412E-04	8.87
rs864003384	18746919	1.412E-04	8.87
rs863555760	18752404	5.161E-05	9.87
rs863293163	18755881	4.227E-02	3.16
rs863691131	18756307	5.320E-03	5.24
rs864181485	18757599	7.067E-04	7.25
rs864214258	18763582	1.762E-01	1.74
rs864246016	18764397	2.708E-04	8.21
rs863552849	18764947	3.336E-03	5.70
rs863955128	18769453	9.221E-03	4.69
ctg7180001328514_773_SGT	18782635	4.834E-02	3.03
rs864252727	18782806	9.822E-01	0.02
rs863360388	18785547	9.822E-01	0.02
rs863548835	18787338	1.005E-02	4.60
rs863407086	18788399	1.005E-02	4.60

rs864209010	18788474	2.122E-01	1.55
rs863531362	18789091	1.005E-02	4.60
rs863387654	18789510	1.005E-02	4.60
rs863986381	18794987	1.461E-01	1.92
rs863739822	18801802	1.005E-02	4.60
ctg7180001744125_623_SCT	18823705	1.200E-02	4.42
rs864138462	18827172	2.724E-01	1.30
rs864148002	18828430	7.029E-01	0.35
rs863448305	18837393	6.100E-01	0.49
rs864170707	18837668	6.100E-01	0.49
rs863918410	18837868	7.088E-01	0.34
rs863486915	18838454	5.416E-01	0.61
rs863392682	18841031	4.051E-01	0.90
rs863633225	18845870	8.576E-04	7.06
rs863861104	18846924	8.576E-04	7.06
rs863508427	18850773	1.343E-05	11.22
rs863919979	18850805	1.343E-05	11.22
rs863541055	18851187	2.067E-01	1.58
rs863819255	18864180	5.702E-01	0.56
rs863703516	18865539	1.184E-01	2.13
rs863985900	18866647	1.184E-01	2.13
rs863284245	18866942	1.184E-01	2.13
rs863242467	18872043	1.184E-01	2.13
ctg7180001805095_19769_SCT	18881719	3.959E-03	5.53
rs863836278	18882170	3.691E-01	1.00
rs863981730	18888084	1.184E-01	2.13
rs863918510	18888554	8.522E-01	0.16
rs864029155	18889161	1.184E-01	2.13
rs863229108	18889269	1.184E-01	2.13
rs863320925	18890667	4.504E-05	10.01
rs864098139	18891332	8.707E-01	0.14
rs863291655	18891601	8.707E-01	0.14
ctg7180001805095_14378_SCT	18891696	3.988E-01	0.92
rs863792522	18891905	8.707E-01	0.14
rs864205400	18894521	4.409E-03	5.42
rs863317802	18896131	4.275E-02	3.15
rs864118606	18900481	2.965E-02	3.52
rs863942301	18905202	2.965E-02	3.52
rs863458548	18905614	2.965E-02	3.52
rs863276889	18905782	2.965E-02	3.52
rs863735591	18906569	5.706E-01	0.56
rs864251506	18907256	8.545E-12	25.49
rs863900233	18913428	2.079E-03	6.18

rs863350932	18916170	2.079E-03	6.18
rs863279457	18926749	3.387E-02	3.39
rs863911761	18928361	3.127E-01	1.16
rs864232964	18944506	1.222E-01	2.10
rs864021810	18945934	1.222E-01	2.10
rs863609169	18950641	1.080E-01	2.23
rs864111848	18950916	7.442E-09	18.72
rs863873516	18952527	7.442E-09	18.72
rs863962390	18952568	3.860E-02	3.25
rs863245319	18970280	3.241E-01	1.13
ctg7180001467069_2463_SAC	18972289	3.508E-01	1.05
rs863962816	18987598	3.230E-06	12.64
rs864173761	18987665	3.230E-06	12.64
rs864111198	18987814	8.533E-02	2.46
rs863244357	18988111	8.533E-02	2.46
rs863342759	18989829	1.618E-04	8.73
rs159406129	18990193	1.798E-02	4.02
rs159406129	18990193	1.404E-02	4.27
ctg7180001779364_3569_SAG	18990295	1.231E-02	4.40
rs863725961	18990715	4.842E-02	3.03
rs863579211	18992195	3.531E-02	3.34
rs864108707	18993133	1.231E-02	4.40
rs864083648	18997024	1.708E-04	8.68
rs864029813	18997358	3.531E-02	3.34
rs864104224	18997416	2.227E-01	1.50
rs863603320	19001155	1.405E-04	8.87
rs864222936	19001953	1.405E-04	8.87
rs863798621	19001990	2.134E-06	13.06
rs863455321	19002100	1.405E-04	8.87
rs863305376	19002245	1.405E-04	8.87
rs864170292	19002795	2.155E-06	13.05
rs863313903	19002968	3.531E-02	3.34
rs863278458	19003453	3.531E-02	3.34
rs863756893	19005565	2.141E-04	8.45
rs863415577	19006593	7.171E-07	14.15
rs863396798	19007006	2.157E-05	10.74
rs864135251	19007154	7.171E-07	14.15
rs864113346	19007446	1.980E-06	13.13
rs864208778	19007511	3.531E-02	3.34
rs864208778	19007511	3.531E-02	3.34
BCMO1_81837	19008130	1.708E-04	8.68
BCMO1_81820	19008147	3.531E-02	3.34
rs863908213	19008790	2.917E-06	12.74

rs863289778	19009217	2.119E-06	13.06
rs863613538	19009726	1.632E-03	6.42
rs863831492	19013109	4.694E-06	12.27
BCMO1_SSA26MOD_422424	19017529	4.036E-01	0.91
rs863772928	19018616	3.631E-02	3.32
BCMO1_SSA26MOD_416513	19023616	4.267E-02	3.15
rs863673647	19024187	2.024E-04	8.51
rs863673647	19024187	7.289E-06	11.83
rs863723023	19029542	7.289E-06	11.83
rs864246870	19029622	7.289E-06	11.83
rs863699183	19032982	4.317E-03	5.45
rs863429657	19033176	4.317E-03	5.45
BCMO1_ssa26mod_408753	19033212	3.132E-02	3.46
rs863650429	19033344	6.409E-06	11.96
BCMO1_SSA26MOD_407073	19034892	2.606E-04	8.25
rs863841221	19035282	1.677E-11	24.81
rs863735013	19039889	7.183E-04	7.24
rs864185097	19042770	1.578E-04	8.75
rs864185097	19042770	7.535E-06	11.80
rs863815058	19043542	1.578E-04	8.75
rs863815058	19043542	7.535E-06	11.80
BCMO1_45633	19044104	2.418E-02	3.72
rs863457647	19044305	1.578E-04	8.75
ctg7180001733669_1558_SCT	19047746	3.863E-02	3.25
BCMO1_43314	19048254	1.530E-01	1.88
rs864136920	19049331	1.434E-25	57.20
rs863699640	19050133	1.094E-24	55.17
rs159403238	19050343	1.434E-25	57.20
rs159403238	19050343	1.434E-25	57.20
BCMO1Jan2012_50882	19051049	4.762E-01	0.74
rs863433469	19051075	1.530E-01	1.88
BCMO1Jan2012_50796	19051135	1.179E-01	2.14
rs864240256	19051152	1.201E-01	2.12
ctg7180001733669_5231_SAG	19051419	1.530E-01	1.88
rs863294679	19051426	1.530E-01	1.88
rs863460708	19052008	1.434E-25	57.20
rs863979260	19052145	1.530E-01	1.88
rs864121002	19053125	6.565E-01	0.42
rs863622198	19053298	1.179E-01	2.14
rs864133094	19053468	1.094E-24	55.17
BCMO1_37970	19053598	1.179E-01	2.14
rs864140717	19056436	1.179E-01	2.14
rs863667798	19058667	1.530E-01	1.88

rs863281847	19060211	3.290E-26	58.68
rs863418153	19061809	1.719E-23	52.42
rs863556042	19062953	3.574E-27	60.90
rs863721927	19063311	1.188E-01	2.13
rs863434642	19064216	2.946E-26	58.79
rs863645576	19064952	5.023E-27	60.56
rs863699886	19068122	6.677E-27	60.27
BCMO1_SSA26MOD_361489	19068133	6.677E-27	60.27
rs864032545	19069882	1.887E-27	61.53
rs863388734	19069884	1.426E-28	64.12
rs863560551	19073497	9.160E-30	66.86
rs863946832	19076391	7.168E-01	0.33
rs863532363	19077089	4.815E-27	60.60
rs863554585	19077273	1.107E-34	78.19
rs863442990	19079127	8.095E-54	122.25
rs863867200	19079917	6.768E-35	78.68
rs864103853	19080027	1.196E-09	20.54
rs863785818	19081573	1.377E-53	121.72
rs863641555	19082257	1.503E-11	24.92
rs863758878	19082481	1.503E-11	24.92
BCMO1_3801	19082663	1.153E-29	66.63
BCMO1_3484	19082980	8.757E-24	53.09
rs863682291	19083074	3.877E-11	23.97
BCMO1_3241	19083223	2.259E-02	3.79
rs863940219	19083998	9.841E-03	4.62
BCMO1_1017	19085447	3.895E-01	0.94
rs863480295	19085953	3.895E-01	0.94
rs864104703	19086549	3.920E-01	0.94
rs863997987	19086803	6.487E-01	0.43
rs864163658	19088480	3.540E-16	35.58
rs864239694	19090689	2.229E-01	1.50
rs863356149	19092929	3.540E-16	35.58
rs863242077	19093101	3.540E-16	35.58
rs863837258	19093286	3.368E-01	1.09
rs863560128	19093347	2.642E-06	12.84
ctg7180001866403_1903_SCT	19093415	3.540E-16	35.58
rs864254197	19093698	2.642E-06	12.84
rs864048824	19094438	3.540E-16	35.58
rs863882958	19094726	5.983E-01	0.51
rs864034428	19095060	6.992E-06	11.87
rs864020357	19098534	9.850E-05	9.23
rs863808798	19118915	4.909E-01	0.71
rs863889518	19121606	3.540E-16	35.58

rs863261265	19122801	4.909E-01	0.71
rs863540518	19123299	4.696E-03	5.36
rs863565552	19123704	1.828E-02	4.00
rs864190920	19124146	9.169E-01	0.09
rs863360143	19124890	3.540E-16	35.58
rs863403978	19129310	3.540E-16	35.58
rs864106897	19129402	6.115E-03	5.10
rs863410946	19129698	5.374E-01	0.62
rs863380259	19140354	3.540E-16	35.58
rs863760013	19140509	3.540E-16	35.58
rs863881957	19140703	3.441E-01	1.07
rs863682040	19140768	3.441E-01	1.07
ctg7180001557189_601_SAG	19140919	6.130E-16	35.03
ctg7180001864332_11963_SGT	19140920	6.037E-01	0.50
rs863246299	19141015	2.480E-01	1.39
rs863614786	19141898	4.070E-12	26.23
rs863604110	19142352	4.070E-12	26.23
rs863298115	19146845	3.540E-16	35.58
rs863545275	19148069	3.540E-16	35.58
rs863367735	19151456	3.540E-16	35.58
rs864156133	19151502	3.540E-16	35.58
rs863761804	19151629	3.540E-16	35.58
rs863585018	19152298	3.540E-16	35.58
rs863469162	19152617	3.540E-16	35.58
rs864177234	19153282	3.540E-16	35.58
rs863356450	19160265	3.540E-16	35.58
rs864169181	19161558	3.441E-01	1.07
rs863287840	19161911	3.441E-01	1.07
rs863893068	19167956	8.787E-17	36.97
rs863953675	19168444	3.540E-16	35.58
rs863464930	19168914	2.014E-15	33.84
rs863903779	19170163	3.026E-14	31.13
rs864138670	19172547	1.728E-15	33.99
rs864013762	19176817	3.026E-14	31.13
rs864058885	19185539	1.728E-15	33.99
rs864038949	19187004	7.166E-05	9.54
ctg7180001848181_13203_SAG	19187310	2.035E-01	1.59
rs863907852	19194529	1.728E-15	33.99
rs863582789	19203125	5.492E-01	0.60
rs863977958	19230908	2.021E-03	6.20
rs863388278	19232649	1.794E-01	1.72
rs863821607	19247838	3.157E-01	1.15
rs864031964	19249105	1.965E-01	1.63

rs863430709	19249788	1.965E-01	1.63
rs863965270	19252011	1.965E-01	1.63
rs863575174	19253089	1.965E-01	1.63
rs864050148	19254654	2.069E-03	6.18
rs863541276	19255154	1.965E-01	1.63
rs864168424	19260022	5.198E-04	7.56
rs863538804	19269705	2.291E-09	19.89
ctg7180001902815_11658_SAG	19270046	5.492E-01	0.60
rs863261312	19270596	9.020E-10	20.83
rs863250331	19271060	4.054E-09	19.32
rs864153919	19275782	2.246E-08	17.61
rs864092962	19279098	2.045E-01	1.59
rs863992350	19279255	6.377E-07	14.27
rs863537347	19281062	3.241E-01	1.13
rs864175148	19284305	9.448E-01	0.06
rs864187608	19285401	1.017E-08	18.40
rs863265930	19285976	9.939E-04	6.91
rs863749747	19286936	3.437E-03	5.67
rs863530042	19287078	4.565E-03	5.39
rs863313687	19287560	4.565E-03	5.39
rs863669620	19288417	2.497E-03	5.99
rs863490286	19288985	6.340E-10	21.18
rs863661159	19290159	6.606E-05	9.62
rs863888145	19292315	1.091E-10	22.94
rs863633141	19295827	2.613E-02	3.64
rs864052472	19296862	9.888E-11	23.04
rs863548467	19297779	2.005E-01	1.61
rs863406273	19298897	1.198E-01	2.12
rs864025048	19299502	7.002E-06	11.87
rs864065881	19304485	5.399E-03	5.22
rs864216474	19307972	4.935E-01	0.71
rs863335535	19308404	4.935E-01	0.71
rs864031125	19309772	4.935E-01	0.71
rs864132037	19310772	2.302E-06	12.98
rs863407349	19323334	4.638E-03	5.37
rs863385810	19330797	1.311E-02	4.33
rs863250308	19331271	1.640E-02	4.11
rs863720013	19331308	3.013E-05	10.41
rs864151096	19344468	1.311E-02	4.33
ctg7180001844909_9843_SAG	19355435	4.068E-03	5.50
rs864137208	19355971	9.453E-01	0.06
rs864078587	19356038	5.880E-10	21.25
rs863969489	19356072	5.880E-10	21.25

rs864140305	19361565	5.880E-10	21.25
rs863437066	19366146	5.880E-10	21.25
rs863345275	19366194	2.108E-01	1.56
rs863360863	19369836	2.144E-03	6.15
rs863448879	19371556	8.300E-02	2.49
rs863576794	19371753	8.300E-02	2.49
rs864210114	19371975	8.300E-02	2.49
rs863842708	19372545	8.300E-02	2.49
rs863547918	19372813	3.772E-03	5.58
rs863961201	19374755	9.702E-08	16.15
rs863588898	19375646	9.702E-08	16.15
rs863360001	19376421	6.716E-01	0.40
rs863818733	19377186	5.314E-10	21.36
rs863751556	19377966	6.076E-02	2.80
rs863936459	19391643	2.302E-10	22.19
rs863789196	19402947	1.847E-03	6.29
rs863234380	19403130	2.024E-10	22.32
rs863346344	19406035	1.418E-02	4.26
ctg7180001215171_1534_SGT	19406868	2.024E-10	22.32
rs863772984	19407499	1.227E-06	13.61
rs864171414	19414199	9.079E-01	0.10
rs863977490	19414428	6.272E-03	5.07
rs864117195	19415676	2.162E-04	8.44
rs864092011	19418293	1.512E-14	31.82
rs863338437	19427331	2.140E-04	8.45
rs864097033	19428398	3.857E-02	3.26
rs863241256	19441424	3.980E-05	10.13
rs863708442	19446180	3.399E-03	5.68
rs863578259	19446883	5.221E-04	7.56
